# Clinical experience of treatment of immunocompromised individuals with persistent SARS-CoV-2 infection based on drug resistance mutations determined by genomic analysis: a descriptive study

**DOI:** 10.1186/s12879-023-08797-6

**Published:** 2023-11-09

**Authors:** Haruka Shimazu, Daiki Wada, Shuhei Maruyama, Akira Inoue, Masami Kashihara, Tomoyuki Yoshihara, Fukuki Saito, Kazuhisa Yoshiya, Yasushi Nakamori, Yasuyuki Kuwagata

**Affiliations:** 1 Department of Emergency and Critical Care Medicine, Kansai Medical University General Medical Center, 10-15 Fumizono-Cho, Moriguchi, Osaka 570-8507 Japan; 2 Department of Emergency and Critical Care Medicine, Kansai Medical University Hospital, 2-3-1 Shinmachi, Hirakata, Osaka 573-1191 Japan

**Keywords:** COVID-19, SARS-CoV-2, Immunocompromised, Genomic analysis

## Abstract

**Background:**

The efficacy of antiviral drugs that neutralize antibody drugs and fight against SARS-COV-2 is reported to be attenuated by genetic mutations of the virus in vitro. When B-cell immunocompromised patients are infected with SARS-COV-2, the infection can be prolonged, and genetic mutations can occur during the course of treatment. Therefore, for refractory patients with persistent COVID-19 infection, genomic analysis was performed to obtain data on drug resistance mutations as a reference to determine which antiviral drugs and antibody therapies might be effective in their treatment.

**Methods:**

This was a descriptive analysis with no controls. Patients were diagnosed as having COVID-19, examined, and treated in the Kansai Medical University General Medical Center between January 2022 and January 2023. The subjects of the study were B-cell immunocompromised patients in whom genome analysis of SARS-CoV-2 was performed.

**Results:**

During the study period, 984 patients with COVID-19 were treated at our hospital. Of those, 17 refractory cases underwent genomic analysis. All 17 patients had factors related to immunodeficiency, such as malignant lymphoma or post-organ transplantation. Eleven patients started initial treatment for COVID-19 at our hospital, developed persistent infection, and underwent genomic analysis. Six patients who were initially treated for COVID-19 at other hospitals became persistently infected and were transferred to our hospital. Before COVID-19 treatment, genomic analysis showed no intrahost mutations in the NSP5, the NSP12, and the RBD regions. After COVID-19 treatment, mutations in these regions were found in 12 of 17 cases (71%). Sixteen patients survived the quarantine, but one died of sepsis.

**Conclusions:**

In genomic analysis, more mutations were found to be drug-resistant after COVID-19 treatment than before COVID-19 treatment. Although it was not possible to demonstrate the usefulness of genome analysis for clinical application, the change of the treatment drug with reference to drug resistance indicated by genomic analysis may lead to good outcome of immunocompromised COVID-19 patients.

**Supplementary Information:**

The online version contains supplementary material available at 10.1186/s12879-023-08797-6.

## Background

In November 2021, the B.1.1.529 (Omicron) variant of severe acute respiratory syndrome coronavirus 2 (SARS-CoV-2) was detected in South Africa and since then has rapidly spread around the world. The Omicron variant of SARS-CoV-2 has been divided into five distinct sub-lineages: BA.1, BA.2, BA.3, BA.4, and BA.5. It has been reported that the efficacy of drugs against SARS-CoV-2 is attenuated by genetic mutations of the virus in vitro. The recent emergence of the SARS-CoV-2 Omicron (B.1.1.529 lineage) variants possessing numerous mutations has raised concerns about the decreased effectiveness of current vaccines, therapeutic monoclonal antibodies, and antiviral drugs for COVID-19 against these variants [1].

Several studies have reported that COVID-19 infection is associated with severe disease and high mortality in patients with malignant lymphoma and organ transplantation with B-cell immunodeficiency [2, 3]. Patients with immunosuppression are at risk for prolonged SARS-CoV-2 infection [4]. In several case reports, investigators indicated that multi-mutational SARS-CoV-2 variants can arise during the course of such cases of persistent COVID-19 infection [5–8].

We considered that in immunosuppressed patients, ordinary COVID-19 treatment could not suppress this virus within a normal quarantine period. Therefore, from September 2021, we introduced a novel treatment protocol combining antiviral and neutralizing antibody-based therapies with the monitoring of spike-specific antibodies and viral load for immunocompromised patients with persistent COVID-19 infection [9]. In addition—for refractory cases in particular—a genomic analysis was performed, and data obtained about drug resistance mutations were used as a reference to determine which antiviral drugs and antibody therapies might be effective in the treatment of these patients.

This descriptive, retrospective study of current genomic analysis of specimens from immunocompromised patients with persistent SARS-CoV-2 infection was conducted to examine the status of intrahost gene mutations and drug resistance gene mutations. The clinical courses of three patients treated with reference to the results of the genomic analysis are also presented.

## Methods

### Study design and participants

This was a retrospective, single-center, descriptive study with no controls. Patients were diagnosed as having COVID-19 confirmed by RT-PCR test for SARS-CoV-2 from sputum or nasopharyngeal swab and examined and treated in the Department of Emergency and Critical Care Medicine, Kansai Medical University General Medical Center, Osaka, Japan, between January 2022 and January 2023. The subjects of the study are patients in whom genome analysis of SARS-CoV-2 was performed on specimens before the start of treatment and then again due to persistent infection by the virus, and patients who were treated at other hospitals but were transferred to our hospital due to persistent infection. Persistent infection was defined as immunocompromised cases in which isolation was not ended even if more than 2 weeks had passed since the onset of the disease because anti-SARS-COV-2 therapy was initiated but viral load rebounded or viral load persisted at high levels. Patients aged < 18 years, pregnant women, and patients in cardiopulmonary arrest on admission were excluded.

### Treatment protocol for persistent COVID-19

Neutralizing antibodies were administered to patients with no increase in spike-specific antibody commensurate with the number of vaccinations recieved. Prior to administration of neutralizing antibodies, SARS-CoV-2 variants were identified by single nucleotide polymorphism PCR assays targeting SARS-CoV-2 S-gene mutations L452R, L452Q, ins214EPE, G339D, and N460K. We selected neutralizing antibodies according to the subvariants with reference to previous reports [10–13]. Treatment with sotrovimab was selected for BA.1, imdevimab/casirivimab for BA.2, and imdevimab/casirivimab or tixagevimab/cilgavimab for the BA.5 variant. For patients diagnosed as having persistent viral infection, genomic analysis of SARS-COV-2 was performed and drug selection was based on drug resistance. Antivirals were selected by each physician from among remdesivir, molnupiravir, nirmatrelvir/ritonavir, and ensitrelvir, based on renal function, liver function, concomitant medications, and the patient’s ability to take them orally. Monitoring of viral load during treatment allowed us to evaluate the effects of the antivirals and neutralizing antibody-based therapies and determine when to end treatment. In the initial time before treatment and when viral load did not decrease after treatment, genomic analysis was performed and antiviral drugs were changed with reference to the genetic mutations found for drug resistance. Treatment was terminated when spike-specific antibody increased sufficiently or when the virus was no longer detectable by PCR.

### Measurement of viral load and identification of variants

After RNA extraction (magLEAD 12gC, Precision System Science Co., Ltd., Tokyo, Japan), SARS-CoV-2 was detected by RT-PCR with a SARS-CoV-2 Detection Kit -Multi- (Toyobo, Osaka, Japan) according to the manufacturer’s instructions. The Ct value of RT-PCR was used to calculate the viral load. Single nucleotide polymorphism PCR assays were performed according to the prevalent variants by using specific probes and primers for ins214EPE (Takara Bio Co., Shiga, Japan) and L452R, L452Q, G339D, and N460K (Thermo Fisher Scientific, MA, USA).

### Genome analysis methods

For genomic analysis, RNA extracted for RT-PCR was used. The sequencing run was performed by using the Ion AmpliSeq SARS-CoV-2 Research Panel or the Ion AmpliSeq SARS-CoV-2 Insight Research Assay on a Genexus Integrated Sequencer (ThermoFisher Scientific). The Ion Torrent Genexus Integrated Sequencer is an automated next-generation sequencing system that integrates library preparation, template preparation, sequencing, and data analysis.

### Assessment of genetic mutations within individuals

We used the Outbreak.info web site to count the number of SARS-CoV-2 subvariants registered in the database of the Global Initiative on Sharing All Influenza Data (GISAID) on September 4, 2023 [14]. Intrahost genetic mutations occurring prior to the administration of anti-SARS-CoV-2 drugs were defined as minority mutations (less than 50% in deep sequence). For intrahost genetic mutations occurring after the administration of anti-SARS-CoV-2 drugs, if there was a pre-treatment genomic analysis, intrahost genetic mutations were defined as mutations that were not present in the pre-treatment analysis but newly emerged in the post-treatment genomic analysis and were rare (i.e., less than 1% of the variants were registered in GISAID). In the absence of pre-treatment genomic analysis, intrahost genetic mutations were defined as rare mutations (less than 1% of the variants were registered in GISAID).

For all gene mutations in the nonstructural proteins (NSP)5 region, NSP12 region, and receptor-binding domain (RBD) region of the spike protein, frequencies were counted using the Outbreak.info website. Gene mutations with a frequency of 1% or less are listed by amino acid numbering in the GISAID in Table 1. Genetic mutations associated with drug resistance were searched through PubMed and pharmaceutical company fact sheets, and relevant mutations indicating a drug-resistant mutation noted in a paper or manufacturer’s fact sheet were marked with an asterisk (*) in Table 1.

**Table 1:** Details of patients starting treatment at our hospital (Nos. 1–11) and patients who were transferred after treatment at other hospitals (Nos. 12–17)

			**Mutations before the treatment for COVID-19**				**Mutations after the treatment for COVID-19**			
**No**	**PANGOLIN**	**GISAID count**	**NSP5 (number of registrations) [% of mutation]**	**NSP12 (number of registrations) [% of mutation]**	**RBD (number of registrations) [% of mutation]**	**Drugs administered prior to subsequent GA**	**NSP5 (number of registrations) [% of mutation]**	**NSP12 (number of registrations) [% of mutation]**	**RBD (number of registrations) [% of mutation]**	**Outcome**
1	BA.1.1	1047522	–	–	–	Rem, Nir/Rit Sot	–	A625V(11)[50%]	*P337S(71)[54%] *E340K(228)[100%] F374L(11)[39%]	Ended isolation
2	BA.1.1	1047522	–	–	–	Rem Sot	–	–	*S371F(1817)[78%]	Ended isolation
3	BA.1.1.2	62165	–	–	–	Rem Sot	–	*C799F(3)[72%]	–	Ended isolation
4	BA.2.29	8463	–	–	–	Mol Cas/Imd	–	–	–	Ended isolation
5	BA.5.1	254400	–	–	–	Rem, Mol Nir/Rit Cas/Imd	Q273R(2)[45%]	R457C(12)[44%]	*K444R(638)[46%] *G446S(183)[23%]	Ended isolation
6	BA.5.1	254,400	–	–	–	Mol Cas/Imd	–	I145V(5)[57%] D164N(0)[55%]	*G446S(183)[20%] *G446D(114)[34%] P499S(6)[59%]	Ended isolation
7	BA.5.2.1	302404	–	–	–	Mol Cas/Imd	–	D260N(6)[99%]	V445A(1309)[91%]	Ended isolation
8	BA.5.2.1	302404	–	–	–	Rem, Ens Tix/Cil (before onset)	*M49L(0)[100%]	A529V(1808)[100%] G671S(708)[35%] E796D(7)[64%]	*R346S(220)[33%] *N450D(994)[62%]	Ended isolation
9	BA.5.2.6	12935	–	–	–	Mol, Rem Tix/Cil	–	–	–	Ended isolation
10	BA.5.2.46	155	–	–	–	Ens, Mol	–	–	–	Ended isolation
11	BF.5	85649	–	–	–	Mol Sot	–	–	–	Ended isolation
12	BA.1.1.1	66698				Sot	–	P227S(8)[100%]	*P337H(9)[99%] *S371F(272)[99%] Y508H(1)[100%]	Ended isolation
13	BA.5.2.1	302404				Rem, Mol	–	–	–	Ended isolation
14	BA.5.1	254400				Rem, Nir/Rit Tix/Cil	K90R(1127)[96%]	T246I(749)[99%] *C799Y(6)[96%]	–	Ended isolation
15	BA.5.2.1	302404				Rem Cas/Imd	Q273R(1)[24%]	*V166A(8)[34%] E796K(5)[29%]	L368I(23)[24%] K417H(1)[19%] *G446S(227)[25%]	Ended isolation
16	BA.5.2.1	302404				Rem Tix/Cil	K236R(40)[99%]	V233I(36)[100%] G671S(708)[100%] P918L(143)[99%]	L335S(3)[98%] G339Y(46)[58%] *K444R(726)[96%]	Ended isolation
17	BF.5	85649				Rem	–	E876D(0)[27%]	Y369H(0)[33%] V503F(0)[28%]	Death

*PANGOLIN* Phylogenetic Assignment of Named Global Outbreak Lineages, *GISAID* Global Initiative on Sharing All Influenza Data, *NSP* Non-structural protein, *RBD* Receptor-binding domain, *GA* Genomic analysis, *Rem* Remdesivir, *Nir/Rit* nirmatrelvir/ritonavir, *Sot* Sotrovimab, *Mol* molnupiravir, *Cas/Imd* casirivimab/imdevimab, *Tix/Cil* Tixagevimab/cilgavimab, *Ens* Ensitrelvir

^*^Asterisk (*) indicates a drug-resistant mutation noted in a paper or manufacturer's fact sheet

### Data collection

We collected data on and described patient characteristics, comorbidities related to immunodeficiency, spike-specific antibody, vaccination frequency, viral load (initial genomic analysis and subsequent analysis), current immunosuppressive drugs, severity of COVID-19, and the contents of antiviral and neutralizing antibody-based therapy up to the subsequent genomic analysis. In case presentations in which drug resistance gene mutations were suspected, information on viral load, anti-S antibodies, changes over time in sialylated carbohydrate antigen KL-6 (KL-6) as a marker of lung damage [15], and drug administration for SARS-COV-2 were extracted from the medical records.

## Results

### Study subjects

During the study period, 984 patients with COVID-19 were treated at our hospital. Of those, 17 refractory cases underwent genomic analysis. All 17 patients had factors related to immunodeficiency, such as malignant lymphoma or post-organ transplantation (Table 2). Eleven patients started initial treatment for COVID-19 at our hospital, developed persistent infection, and underwent genomic analysis. Six patients who were initially treated for COVID-19 at other hospitals became persistently infected and were transferred to our hospital. Sixteen patients survived the quarantine, but one died of sepsis due to *Candida albicans*.

**Table 2:** Patient characteristics

No	Age	Sex	Comorbidities	Vaccination frequency	Initial spike-specific antibody (U/ml)	Viral load Initial GA (copy/µL) (Days after onset)	Viral load Subsequent GA (copy/µL) (Days after onset)	Current immunosuppressive drug	Severity of COVID-19
1	81	Female	DLBCL	3	61.9	387834 (2)	318187 (31) 218663 (51)	Rituximab Obinutuzumab Bendamustine	Severe
2	71	Male	Kidney transplantation	3	<0.4	1351495 (9)	1466 (24)	Tacrolimus Mycophenolate mofetil Methylprednisolone	Critical
3	66	Female	Kidney transplantation	3	< 0.4	8570 (1)	7670 (21)	Tacrolimus Mycophenolate mofetil Methylprednisolone	Moderate
4	44	Male	Kidney transplantation	3	<0.4	135269 (2)	21798 (19)	Tacrolimus Mycophenolate mofetil Methylprednisolone	Moderate
5	80	Female	DLBCL	2	14.9	2839493 (0)	5853 (13) 2130 (28)	Polatuzumab vedotin Rituximab Bendamustine	Moderate
6	35	Female	Malignant lymphoma	none	20763	2420,183 (3)	627186 (10)	Unknown	Moderate
7	74	Male	Multiple myeloma	none	1.73	5425 (18)	708919 (32)	Pomalidomide Bortezomib	Severe
8	78	Male	Follicular lymphoma	5	1740	34647 (0)	165652 (18)	Polatuzumab vedotin Rituximab Bendamustine	Critical
9	70	Female	Autoimmune hepatitis	none	< 0.4	704487 (6)	54144 (29)	Rituximab Prednisolone	Severe
10	80	Male	Multiple myeloma	4	< 0.4	22116 (0)	2049 (33) 32566 (46)	Pomalidomide Ixazomib	Severe
11	49	Female	Kidney transplantation	3	<0.4	5578198 (2)	842 (19)	Mycophenolate mofetil Cyclosporine	Moderate
12	79	Female	Follicular lymphoma	3	8945		513 (90)	Rituximab Obinutuzumab Bendamustine	Severe
13	54	Male	Follicular lymphoma	3	<0.4		52051 (55)	Rituximab Obinutuzumab Bendamustine	Severe
14	81	Male	Follicular lymphoma	2	2814		537627 (74)	Rituximab Obinutuzumab Bendamustine	Severe
15	81	Male	MALT lymphoma	4	84.5		31406 (173)	Rituximab Bendamustine	Critical
16	54	Female	Follicular lymphoma	none	2153		619 (125)	Obinutuzumab Bendamustine	Severe
17	82	Male	Kidney transplantation	4	<0.4		16042 (17)	Tacrolimus Mycophenolate mofetil Prednisolone	Critical

*GA* Genomic analysis, *DLBCL* Diffuse large B-cell lymphoma, *MALT* Mucosa-associated lymphoid tissue

### Genetic mutations before initiation of COVID-19 treatment

Initial results of genome analysis are listed in the “Mutations before the treatment for COVID-19” column in Table 1. In the NSP5, the NSP12, and the RBD region, no less frequent and minority mutations were found. (Table 1).

### Genetic mutations after COVID-19 treatment and during persistent infection

Multiple results of genome analysis are listed together in the “Mutations after the treatment for COVID-19” column in Table 1. In the NSP 5 region, less frequent mutations were found in 5 of 17 (29%) cases, and drug resistance gene mutations was observed in a patient. In the NSP12 region, less frequent mutations were found in 11 of 17 (65%) cases, and drug resistance gene mutations were observed in three patients. In the RBD region, less frequent mutations were found in 10 of 17 (59%) cases, and drug resistance gene mutations were observed in eight patients and 13 locations. After COVID-19 treatment, mutations were found in 12 of 17 cases (71%) (Table 1).

## Case presentations

### Case no. 1 (follicular lymphoma)

**Fig. 1:**
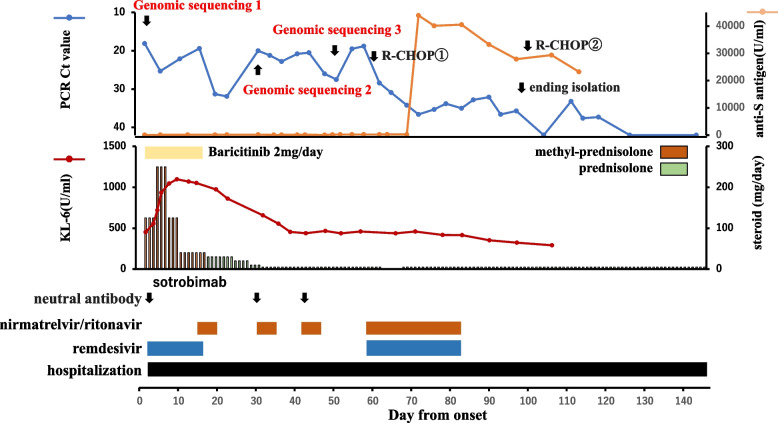
Clinical course of case 1

The patient was an 81-year-old woman who was diagnosed as having Stage IV follicular lymphoma in November 2020 and was treated with six courses of obinutuzumab + bendamustine, followed by rituximab maintenance therapy (Fig. 1). She was taking iguratimod (50 mg/day), prednisone (5 mg/day), and tacrolimus (1 mg/day). She developed COVID-19 with dyspnea in February 2022, 33 days after the last administration of rituximab. On day 2, during a routine visit, her SpO_2_ value decreased to 84% on room air; chest computed tomography (CT) revealed interstitial pneumonia, and RT-PCR was positive (Ct value, 18.1; viral load, 512,873 copies/µl). She was at high risk for severe COVID-19 and was hospitalized and treated with remdesivir and sotrovimab. Genomic sequencing identified BA.1.1. Her interstitial pneumonia gradually improved with methylprednisolone and baricitinib. As her viral load did not decrease after the administration of remdesivir, nirmatrelvir/ritonavir was administered. When nirmatrelvir/ritonavir was terminated, her viral load rebounded, so nirmatrelvir/ritonavir and sotrovimab were re-administered. At that time, her lymphoma worsened and respiratory distress appeared due to enlarged cervical and mediastinal lymph nodes. A genomic analysis was performed again using samples obtained on the 29th day and 49th day. Three new mutations (P337S, E340K, and F374L) were found in the RBD region that were not found in the initial genome analysis. Of the approximately 1.04 million BA.1.1 cases registered in GISAID, these mutations were detected in 71, 228, and 11 cases, respectively. The S371F mutation, which had a frequency of 70% in the initial genomic analysis, increased in frequency to 100% in the subsequent genomic analysis. Although this mutation does not meet the definition of a minority mutation, the possibility of an intrahost mutation cannot be ruled out since only 0.17% was registered in BA.1.1 (Additional file 1). P337S, E340K, and S371F mutation has been reported to confer resistance to sotrovimab [16, 17]. The A625V mutation was found in the NSP12 region with no reported drug resistance-associated mutations. In the NSP5 region, two genomic analyses showed no changes. Nirmatrelvir/ritonavir and remdesivir were administered for COVID-19, while the R-CHOP (rituximab + cyclophosphamide + hydroxydaunorubicin + oncovin + prednisone) regimen was administered for malignant lymphoma. The respiratory distress associated with the patient’s enlarged lymph nodes subsequently improved, her viral load decreased, and the patient’s isolation was ended on day 97. The rebound in viral load was monitored thereafter, and on day 143, RT-PCR was negative for the second consecutive time.

### Case no. 15 (MALT [mucosa-associated lymphoid tissue] lymphoma)

**Fig. 2:**
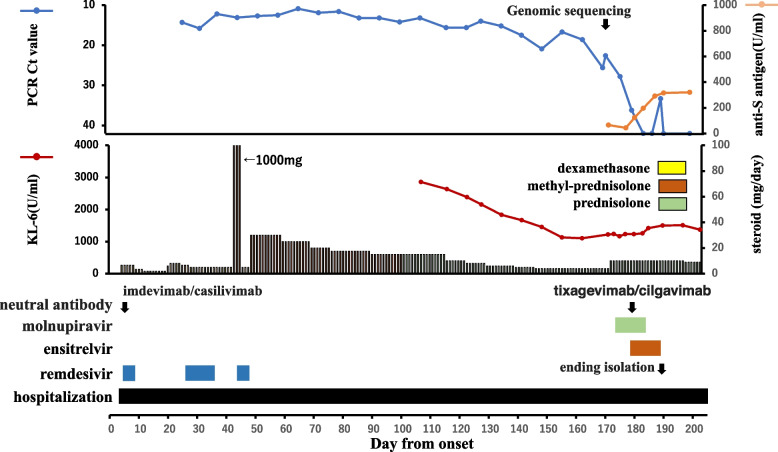
Clinical course of case 16

The patient was an 81-year-old man who was diagnosed as having Stage IV MALT lymphoma in June 2011 and was treated with eight courses of rituximab, followed by rituximab maintenance therapy (Fig. 2). In 2019, rituximab and radiation therapy were started due to mediastinal recurrence, followed by obinutuzumab + bendamustine and rituximab + lenalidomide. He developed COVID-19 with fever in September 2022, 4 days after the last administration of rituximab. On day 3, he was admitted to another hospital where he was treated with remdesivir, dexamethasone, and imdevimab/casirivimab. On day 25, invasive ventilation was started due to worsening interstitial pneumonia. He continued to receive intermittent remdesivir and continuous dexamethasone; however, his pneumonia did not improve, and tracheostomy was performed on day 66. On the 166th day, he was finally weaned from the ventilator; however, he was transferred to our hospital because RT-PCR of nasopharyngeal swab samples continued to detect high viral loads. On admission, RT-PCR was positive for SARS-CoV-2 (Ct value, 22.6; viral load, 31,406 copies/µl). Genomic sequencing identified BA.5.2.1. A G446S mutation was observed in the RBD region. This mutation, which has been reported to confer resistance to imdevimab [18], was found in only 227 of the approximately 300,000 cases with BA.5.2.1 registered in GISAID. In the NSP12 region, in addition to the mutations generally detected in BA.5.2.1, we found a V166A mutation, which was found in eight cases of BA.5.2.1 in GISAID. Mutations in V166A have been reported to confer resistance to remdesivir [19]. After the administration of ensitrelvir, molnupiravir, and tixagevimab/cilgavimab, the virus decreased steadily, and the patient’s isolation was ended on day 188.

### Case no. 16 (follicular lymphoma)

**Fig. 3:**
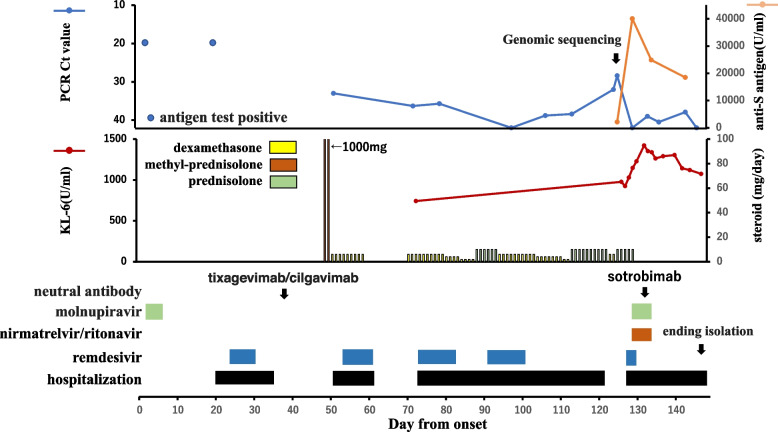
Clinical course of case 17

The patient was a 54-year-old woman who was diagnosed as having Stage IV follicular lymphoma in March 2021 and was treated with six courses of obinutuzumab + bendamustine, followed by obinutuzumab maintenance therapy (Fig. 3). She developed COVID-19 with a sore throat in November 2022, 6 months after the last administration of obinutuzumab. On day 2, she was prescribed molnupiravir for five days. After persistent positive antigen tests, she was admitted to another hospital on day 22 at which she was treated with remdesivir for seven days. On day 38, tixagevimab/cilgavimab was administered after discharge. On day 51, she visited another hospital due to the onset of respiratory distress and was admitted because CT showed extensive interstitial pneumonia. RT-PCR of nasopharyngeal swab samples was negative for SARS-CoV-2, but that of a sputum sample was positive. Remdesivir and dexamethasone were administered for 10 days. After discharge, she was readmitted to the hospital after her dyspnea worsened again, and remdesivir and dexamethasone were resumed. However, her symptoms and CT findings of pneumonia continued to worsen, and she was transferred to our hospital on day 127. On admission, RT-PCR of nasopharyngeal swab samples was positive for SARS-CoV-2 (Ct value, 28.4; viral load, 619 copies/µl). Genomic sequencing identified BA.5.2.1. A K444R mutation was observed in the RBD region. This mutation, which has been reported to confer resistance to cilgavimab [16], was found in only 726 of the approximately 300,000 cases of BA.5.2.1 registered in GISAID. In the NSP12 region, in addition to the mutations generally detected in BA.5.2.1, we detected V233I, G671S and P918L mutations. We could not find any reports of these mutations affecting resistance to remdesivir. After five days of administration of nirmatrelvir/ritonavir, molnupiravir, and sotrovimab, the virus was no longer detectable, and the patient’s isolation was ended on day 138.

## Discussion

It is now generally known that immunocompromised patients, such as those with malignant lymphoma and post-organ transplant patients, are vulnerable to infection with SARS-CoV-2 and have an increased risk of developing severe COVID-19. Although no evidence-based standard of care has been presented, in clinical practice, convalescent plasma, neutralizing antibodies, antiviral drugs, and even combinations of these have been tried [20–22]. We have also experienced patients with malignant lymphoma who have persistent infection and continued serologic negativity in spike-specific antibody test despite long-term administration of antiviral therapy. Therefore, for severely immunocompromised COVID-19 patients, we have used combination therapy with neutralizing antibodies and antiviral drugs [9]. However, we experienced patients with persistent COVID-19 in which their viral load did not decrease or even rebounded after combination therapy. Suspecting drug-resistance mutations, we performed genomic analysis on pre- and post-therapy specimens.

Remdesivir interferes with the SARS-CoV-2 RNA-dependent RNA polymerase (NSP12) [23]. Genetic mutations in the NSP12 region, including V166, N198, S759, V792, C799, and E802, have been reported to reduce the efficacy of remdesivir in vitro [19, 24]. Clinical data from patients with B-cell immunodeficiency with persistent SARS-CoV-2 infection also show that the V166 and E802 mutations are involved in drug resistance after treatment with remdesivir [25, 26]. Remdesivir is the first choice of treatment, especially for patients with pneumonia. Eleven of the patients presented in this report were started on remdesivir. After treatment with remdesivir, eight patients showed new mutations in the NSP12 region, and three showed mutations previously reported to cause drug resistance to remdesivir.

Molnupiravir is phosphorylated to the active form and can act as an alternate and competitive substrate for the SARS-CoV-2 RNA-dependent RNA polymerase, increasing viral RNA replication errors and inhibiting viral replication [27]. Because of this mechanism of action, molnupiravir induces a number of genetic mutations after treatment; however, it has not been reported to lead to drug resistance [28, 29]. In the present study, nine patients also received molnupiravir, but only three showed new mutations in the NSP12 region, which was lower than the number of gene mutations occurring after remdesivir administration.

Nirmatrelvir/ritonavir and ensitrelvir target the 3CL-protease (NSP5) [30, 31]. In vitro data suggest that mutations such as T21, M49, S144, M165, E166, H172, and Q192 in the NSP5 region are involved in drug resistance to 3CL-protease inhibitors [32–34]. Our search of the relevant literature revealed no papers reporting clinical data on nirmatrelvir/ritonavir and ensitrelvir resistance. Although only five patients in this study received 3CL protease inhibitors, M49L mutation, which has been implicated in drug resistance to ensitrelvir, was observed in a patient who was treated with remdesivir followed by ensitrelvir.

Neutralizing antibody drugs against SARS-CoV-2 are effective by binding to the spike protein and inhibiting its binding to the ACE receptor. Therefore, it is strongly affected by mutations in the RBD of the spike protein. Imdevimab/casirivimab, sotrovimab, and tixagevimab/cilgavimab have been approved for use in Japan. These drugs are effective against the B.1.1.7 and B.1.617.2 variants, which were prevalent in Japan before the outbreak of Omicron (B.1.1.529). After the outbreak of Omicron, the effect changed depending on the subvariant. We have selected neutralizing antibodies according to the subvariants with reference to reports of neutralizing activity using pseudoviruses [10–13]. However, additional specific single nucleotide polymorphisms in subvariants have been reported to reduce neutralizing antibody activity [16]. In SARS-CoV-2-infected patients with B-cell immunodeficiency, sotrovimab treatment was reported to result in sotrovimab-resistant single nucleotide polymorphisms (P337 and E340) in the clinical setting [35]. When patients with B-cell immunodeficiency become infected with SARS-CoV-2, the infection may persist despite the administration of antiviral drugs and neutralizing antibodies, and drug resistance due to genetic mutations may be involved in this phenomenon. In the present study, five patients received sotrovimab, five received imdevimab/casirivimab, and four received cilgavimab/tixagevimab. After treatment with sotrovimab, mutations were observed in E337, E340, and S371, close to the binding site of sotrovimab, and after treatment with imdevimab/casirivimab, mutations were observed in K444 and G446, close to the binding site of imdevimab. After administration of cilgavimab/tixagevimab, mutations were observed in R346 and K444, which have been implicated in tixagevimab drug resistance. In patients with B-cell immunodeficiency, if the virus did not disappear promptly after administration of neutralizing antibodies with neutralizing activity against the variants, such escape mutations could be expected to accumulate.

We presented three cases with a typical clinical course of persistent SARS-CoV-2 infection. In these cases, neutralizing antibodies were administered followed by continuation of antivirals, but viral load increased when the antivirals were discontinued. During the course of prolonged infection, the virus developed drug-resistance mutations, especially to neutralizing antibody drugs and remdesivir. In this analysis, although it was not possible to demonstrate the usefulness of genome analysis for clinical application, we changed the treatment drug with reference to the drug resistance indicated by genomic analysis and continued treatment until the RT-PCR test confirmed the virus to be negative. Except for the patient in Case 17, who died of sepsis due to *C. albicans*, we were able to complete the treatment of all patients and end their isolation.

The main limitation of this study is the lack of sufficient evidence to support the efficacy of these monoclonal antibodies and antiviral drugs administered in the treatment of patients infected with COVID-19. There are no trials or rigorous research that includes randomization and larger sample sizes to show the efficacy of neutralizing antibodies as a therapeutic agent. As well, there are no clinical reports on the application of the results of genomic analyses in the selection and determination of antiviral or neutralizing antibody drugs for patients with B-cell immunodeficiency and refractory SARS-COV-2 infection. Further, there is no rationale for the definition of possible intrahost genetic mutations, no evidence to define the frequency of intrahost genetic mutations, nor any consensus regarding the definition and timeline associated with the persistent infection. Although we have experienced cases in which drug resistance information from a genomic analysis was applied in the treatment of COVID-19, further research is needed to accurately determine the effectiveness of this strategy.

## Conclusions

In genomic analysis, more mutations were found to be drug resistant after treatment for COVID-19 than before treatment for COVID-19. Although it was not possible to demonstrate the usefulness of genome analysis for clinical application, changing the treatment drug with reference to drug resistance indicated by genomic analysis may lead to a good outcome in immunocompromised patients with COVID-19.

### Supplementary Information

**Additional file 1.** Whole genome sequencing data of the 31 SARS-CoV-2 samples in our study.

## Data Availability

All relevant data are within the paper and its Additional Information files. The 31 SARS-CoV-2 strains sequences from 17 patients obtained in this study were submitted to the DDBJ (DNA Data Bank of Japan). The corresponding information about strains is resumed in Additional file 1. For sequences whose mutations were not reflected in the FASTA file due to low mutation rates, BAM files were also registered in the database.

## Abbreviations

COVID-19: Coronavirus disease 2019
SARS-CoV-2: Severe acute respiratory syndrome coronavirus 2
Ct: RT-PCR cycle threshold
3CL protease: 3C-like protease
KL-6: Sialylated carbohydrate antigen KL-6
MALT lymphoma: Mucosa-associated lymphoid tissue lymphoma
GISAID: Global Initiative on Sharing All Influenza Data
NSP: Non-structural protein
RBD: Receptor-binding domain
R-CHOP: Rituximab + cyclophosphamide + hydroxydaunorubicin + oncovin + prednisone
